# Formation of Fibrils by the Periplasmic Molecular Chaperone HdeB from *Escherichia coli*

**DOI:** 10.3390/ijms232113243

**Published:** 2022-10-31

**Authors:** Yui Nakata, Yuuto Kitazaki, Hitomi Kanaoka, Erika Shingen, Rina Uehara, Kunihiro Hongo, Yasushi Kawata, Tomohiro Mizobata

**Affiliations:** 1 Department of Chemistry and Biotechnology, Tottori University, Tottori 680-8552, Japan; 2 Course of Biotechnology, Graduate School of Sustainable Social Sciences, Tottori University, Tottori 680-8552, Japan; 3 Center for Green Sustainable Chemistry, Faculty of Engineering, Tottori University, Tottori 680-8552, Japan

**Keywords:** molecular chaperone, amyloid fibril, periplasm, denaturation, HdeB

## Abstract

The molecular chaperones HdeA and HdeB of the *Escherichia coli (E. coli)* periplasm protect client proteins from acid denaturation through a unique mechanism that utilizes their acid denatured states to bind clients. We previously demonstrated that the active, acid-denatured form of HdeA is also prone to forming inactive, amyloid fibril-like aggregates in a pH-dependent, reversible manner. In this study, we report that HdeB also displays a similar tendency to form fibrils at low pH. HdeB fibrils were observed at pH < 3 in the presence of NaCl. Similar to HdeA, HdeB fibrils could be resolubilized by a simple shift to neutral pH. In the case of HdeB, however, we found that after extended incubation at low pH, HdeB fibrils were converted into a form that could not resolubilize at pH 7. Fresh fibrils seeded from these “transformed” fibrils were also incapable of resolubilizing at pH 7, suggesting that the transition from reversible to irreversible fibrils involved a specific conformational change that was transmissible through fibril seeds. Analyses of fibril secondary structure indicated that HdeB fibrils retained significant alpha helical content regardless of the conditions under which fibrils were formed. Fibrils that were formed from HdeB that had been treated to remove its intrinsic disulfide bond also were incapable of resolubilizing at pH 7, suggesting that certain residual structures that are retained in acid-denatured HdeB are important for this protein to recover its soluble state from the fibril form.

## 1. Introduction

The numerous proteins in the *E. coli* periplasm, responsible for vital functions such as the transport of molecules to and from the cell interior, are regularly subjected to rapid and extreme changes in environment, owing to the porous nature of the outer membrane. When an *E. coli* cell is introduced into the stomach of a host, the strongly acidic nature of the stomach is reflected in the periplasmic space, triggering denaturation and loss of activity of important periplasmic proteins. *E. coli* protects itself from the consequences of such protein denaturation events by expressing a pair of small molecular chaperones, HdeA and HdeB, that protect these proteins from irreversible denaturation [1,2].

The amino acid sequence homology between the mature forms of HdeA (89 amino acid residues) and HdeB (79 amino acid residues) [3] is relatively low, with only 11 residues that are regarded as identical, and an overall sequence homology below 20%. However, the subunit structure of these two polypeptides, α-helical rich subunits with a single disulfide bond (HdeA, between Cys18 and Cys66 [1]; HdeB, between Cys10 and Cys58 [4]) are nearly superimposable [5], suggesting perhaps a convergence in subunit structure from divergent sequences. At pH 7, both chaperones form stable dimers [1,5,6]. Notably, these native dimers of HdeA and HdeB are inactive as molecular chaperones. To protect their respective clients at acidic pH [7], both proteins first undergo acid-triggered structural changes to form dynamic conformations that then recognize and bind to denatured clients. In the case of HdeA, strongly acidic conditions (pH < 3) trigger the dissociation of the dimer and the exposure of the dimer interface that facilitates substrate protein recognition [8], which activates the molecular chaperone activity seen for HdeA at pH < 3 [9,10,11]. In contrast, for HdeB, moderately acidic pH (pH < 7 to pH 4) conditions induce subtle changes in the structural characteristics of the native dimer that facilitates substrate recognition and binding [4,12]. Transition to a lower pH (pH < 3), however, further denatures HdeB, induces dissociation of the dimer, and abolishes its molecular chaperone activity. Therefore, there is a distinct pH range where HdeB is active as a molecular chaperone (estimated as between pH 4~5, [12]). Through these differences in pH-induced structural dynamics, HdeA and HdeB utilize subtly different molecular mechanisms to complement each other in providing protection to the cell against a wide range of acidic conditions [2]. Upon return to neutral pH, the two molecular chaperones refold into their inactive, native dimers, releasing their clients and becoming dormant. This reversible nature of HdeA and HdeB acid denaturation allows these two molecular chaperones to use it as a form of environmental sensor that responds to changes in the acidity of the periplasm and assists protein clients only when required.

The dynamic and unstable nature of the active acid denatured form of HdeA prompted us to consider if this state could be capable of forming alternate structures under the same low pH conditions. We subsequently performed in vitro experiments which demonstrated that acid denatured HdeA, upon mild shaking, could form regular fibrillar aggregates that were very similar to the amyloid fibrils that could be observed in cells of patients afflicted with neurodegenerative diseases such as Parkinson’s disease and Alzheimer’s disease [13]. The fibril form of HdeA, however, displayed two unusual characteristics that are not generally seen in the fibrils associated with the above diseases; first, HdeA fibrils that are formed at low pH readily resolubilize and revert to the original soluble native dimer upon a simple shift to neutral pH, and second, HdeA fibrils retained a significant fraction of α-helical content, suggestive of a partial preservation of the original secondary structure. The reversible nature of the fibrils formed suggested that in HdeA, a robust folding landscape exists where HdeA freely converts between an inert native state, an active denatured state, and an inactive, insoluble fibril depending on the experimental pH.

We were intrigued by this structural dynamism seen in HdeA and its ability to assume many structural forms interchangeably and considered the possibility that HdeB, which utilizes a similar molecular mechanism to achieve chaperone function, might also show a similar structural versatility. To test this idea, we performed in vitro experiments on *E. coli* HdeB at low pH to probe its tendencies to form fibrillar aggregates. Our experiments revealed that HdeB indeed tended to form regular fibril structures under acidic conditions, and these fibrils, like those of HdeA, could be resolubilized by a simple shift to pH 7. However, we noted some differences unique to HdeB, which hinted at a significantly more complex structural landscape compared to HdeA.

We present here our findings on the pH-dependent fibrillation of the periplasmic molecular chaperone HdeB from *E. coli*. A prominent characteristic that diverged from the behavior seen for the analog HdeA was the discovery of multiple experimental conditions that led to the formation of “irreversible” fibril forms that could not be resolubilized at pH 7. In the case of HdeB, the tendency to form fibrils was unrelated to its molecular chaperone activities, as the pH regions where fibrils and molecular chaperone activities were, respectively, observed are different. We discuss the structural implications that are brought to light when we compare the fibril forming reactions of these two periplasmic molecular chaperones, as well as the relevance of these characteristics to the general question of protein structural dynamism.

## 2. Results

### 2.1. Fibrillation of HdeB Proceeds at Low pH from an Acid Denatured State

**Figure 1:**
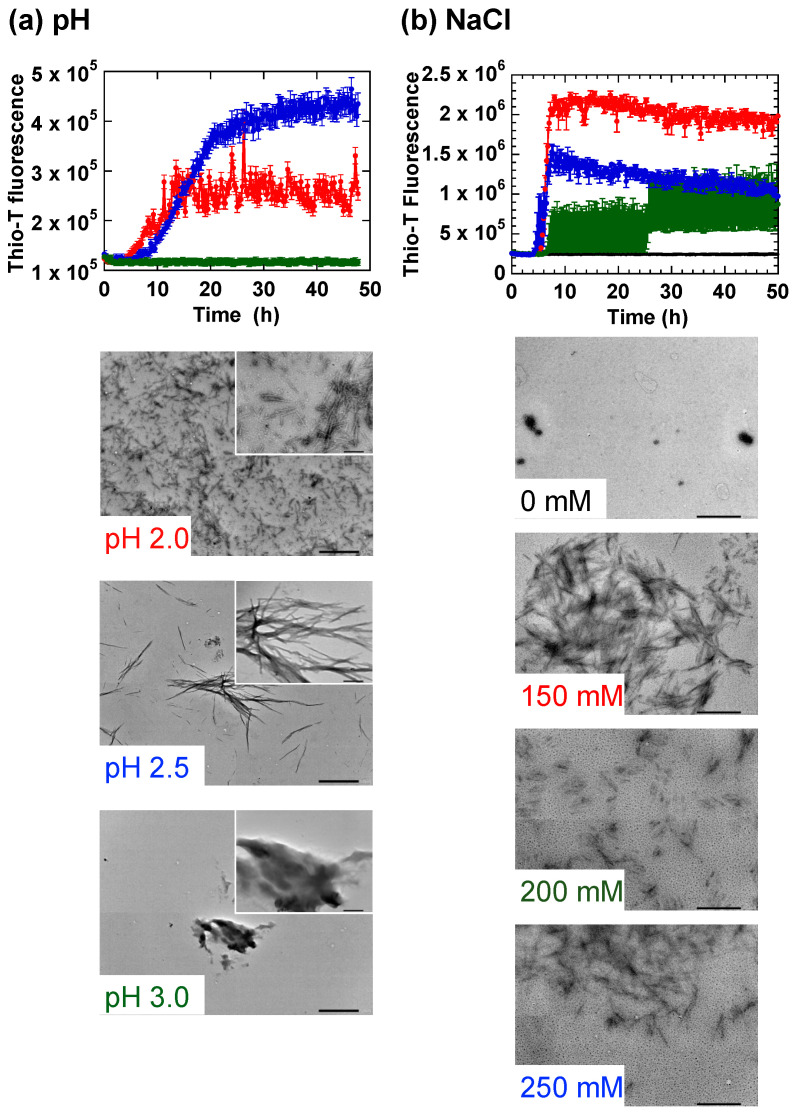
Formation of fibrillar aggregates by HdeB. (**a**) Dependence of fibrillation on experimental pH. Samples of purified HdeB were incubated in Glycine-HCl (Gly-HCl) buffer adjusted to the indicated pH values. NaCl was added to a concentration of 250 mM in each sample. The data are averages ± standard errors of three separate samples measured. TEM micrographs of representative samples are shown below the time course panel (the colors of the legends in the inset are matched to the color of the traces in the time course). In each TEM image, the larger panel is an image obtained at a magnification of ×8000 (the scale bar in the lower right corner = 1.0 µm), and the inset is a magnified image of the larger panel obtained at a magnification of ×40,000 (scale bar: 200 nm). (**b**) Dependence of fibrillation on the concentration of NaCl. HdeB was incubated with agitation in Gly-HCl buffer adjusted to pH 2.0 with the indicated concentrations of NaCl added. Data shown are averages ± standard errors derived from three measurements. Below the time course panel, representative TEM images of each sample obtained at a magnification of ×20,000 is shown (scale bar; 500 nm). The amount of fibrils that could be observed in TEM images for each salt concentration correlated roughly with the Thioflavin-T (Thio-T) fluorescence signal observed in the time course experiments.

We first probed the experimental conditions under which HdeB tended to form fibrils. Figure 1 shows the in vitro fibrillation of HdeB monitored under a variety of conditions, focusing mainly upon pH and NaCl concentration conditions. We found that, as was the case for HdeA, acid denatured HdeB could also be induced to form fibrils upon gentle shaking. HdeB fibrils were only observed at pH values below 3.0 (Figure 1a, the concentration of NaCl was 250 mM, c.f. Figure 1b). Compared to reactions at pH 2.0, fibrillation at pH 2.5 was characterized by an extended lag interval and a stronger fluorescence signal. When the morphologies of the aggregates formed were observed using transmission electron microscopy (TEM), images showed that at pH 2.0, HdeB tended to form clumps consisting of numerous short fibrils, and at pH 2.5, longer fibrils were more prevalent. At pH 3.0, amorphous aggregates were the main forms observed, which was consistent with the lack of a Thio-T signal. Next, we probed the fibrillation of HdeB at pH 2 in the presence of varying concentrations of NaCl. We observed that HdeB fibrils were observed only in the presence of moderate to high NaCl concentrations (higher than 150 mM), suggesting that a certain minimal salt concentration was required for fibrillation to occur. The results in Figure 1 confirmed that HdeB also could form regular fibrillar aggregates from its acid-denatured state in a manner similar to HdeA. Interestingly, the pH at which HdeB fibrils tended to form (pH < 3) was different from the pH range at which HdeB displays molecular chaperone activity (pH 4~5, [12]) and matched the pH region where the dimeric structure of HdeB would be lost [5,12]. In the case of HdeB therefore, a more extensively denatured molecule tended to form fibrils, and this form was decoupled from the molecular chaperone activity of this polypeptide. This characteristic was different from HdeA, in which the fibril forming conformation shared common structural characteristics with the form that expressed molecular chaperone activities.

### 2.2. Prolonged Incubation of HdeB Fibrils at pH 2 Converts Fibrils into an Irreversible Form

With HdeA, fibrils formed at low pH could be easily resolubilized at neutral pH, reflecting the robust refolding tendencies of HdeA regarding acid denaturation and amyloid formation [13]. To see if HdeB, which utilizes a similar reversible denaturation mechanism to modulate its molecular chaperone activity, shared a similar tendency, we probed the effects of shifting the experimental pH to 7 after fibrils had been formed at pH 2.

**Figure 2:**
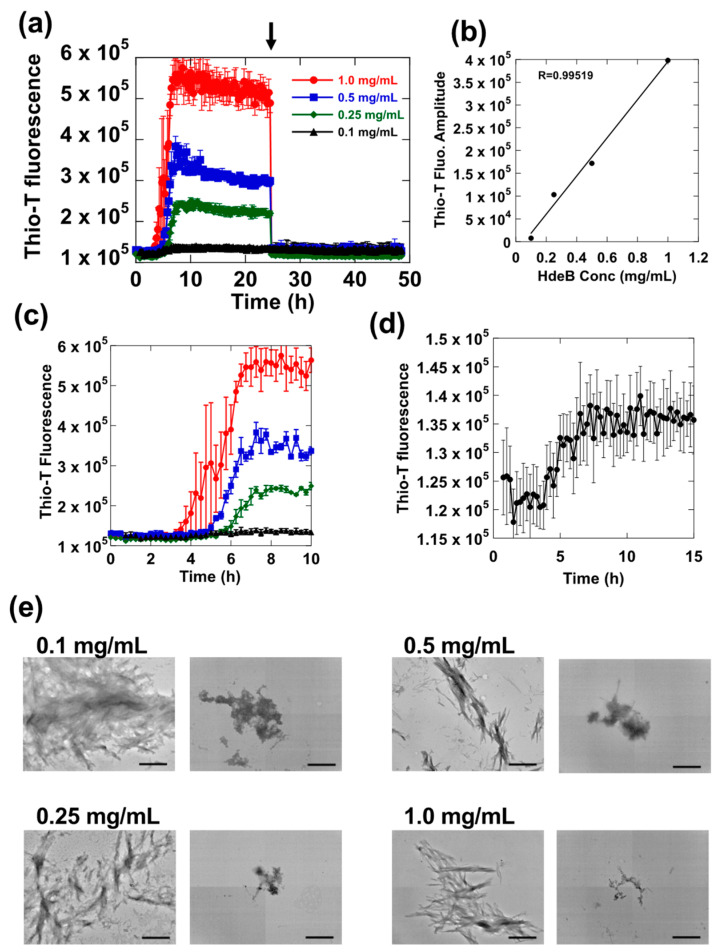
Fibrillation of HdeB at different concentrations. Samples of HdeB in various concentrations ranging from 0.1 mg/mL to 1.0 mg/mL were incubated with shaking in 20 mM Gly-HCl, pH 2.0, containing 250 mM NaCl. Thio-T fluorescence was monitored periodically to obtain the fluorescence traces shown in panel (**a**). The pH of each sample was shifted to 7 with addition of Tris base at the time depicted by the arrow. Panels (**b**) through (**d**) are derived from the initial data shown in (**a**); panel (**b**) shows the dependence of the net Thio-T fluorescence change during the fibril-forming reaction (derived by subjecting the raw fluorescence value at t = 0 from the fluorescence value at t = 24 h of each sample) on the HdeB concentration. Panel (**c**) is an expanded view of the first 10 h of the assay that facilitates observation of changes in lag time for each sample. Panel (**d**) is a magnified view of the 0.1 mg/mL HdeB sample, which was difficult to discern in the traces in panels (**a**,**d**). Panel (**e**); TEM images taken from each sample before and after the shift to pH 7, denoted by the arrow in panel (**a**). Magnification of the images were fixed at ×20,000, and the scale bar denotes 500 nm.

As seen in Figure 2, we observed that, after fibrils had been formed at pH 2.0 (judging from the plateau in the Thio-T fluorescence signal seen at t >10 h), when we shifted the pH of the reaction at t = 24 h from 2.0 to 7.0 (Figure 2a, arrow), we observed an immediate decrease in the Thio-T fluorescence signal, suggesting that, in a manner similar to HdeA, HdeB fibrils formed at pH 2.0 were indeed resolubilizing at pH 7.0. This reversible fibrillation of HdeB was observed at various different concentrations of HdeB, ranging from a moderately high 1 mg/mL down to 0.1 mg/mL, where changes in Thio-T fluorescence became harder to detect reliably (an expanded plot is depicted in Figure 2d). The net change in Thio-T fluorescence intensity for each sample during the reaction was proportional to the HdeB concentration (Figure 2b), which supported the idea that the Thio-T fluorescence change was reliably reporting on the amount of HdeB fibril formed. Regarding changes in the lag time on HdeB concentration, an expanded abscissa plot of Figure 2a (Figure 2c) showed that at lower HdeB concentrations, there was an increase in the lag time that was also dependent on the HdeB concentration.

Upon a shift in pH to 7, in each sample the Thio-T fluorescence signal decreased rapidly, and TEM analysis of the samples before and after pH shift showed that HdeB fibrils were converted into amorphous aggregates, indicating that the fibrils lose their regular structures. Taken together, the results in Figure 2 reflect a pH dependent fibrillation of HdeB at pH 2, in which the fibrils are deconstructed upon a shift to neutral pH.

During the course of our initial exploration of HdeB fibrillation we quite frequently experienced inconsistencies in the behavior of HdeB, particularly after fibrils had been formed. In some samples, reversibility could be confirmed by fluorescence, while in other cases fibrillar structures could be detected after the pH shift. In order to determine the reasons for these discrepancies, we probed for the existence of various additional experimental parameters that might affect the reversible behavior of HdeB fibrils. In a first look, we performed HdeB fibril experiments where the incubation time at pH 2 was changed.

**Figure 3:**
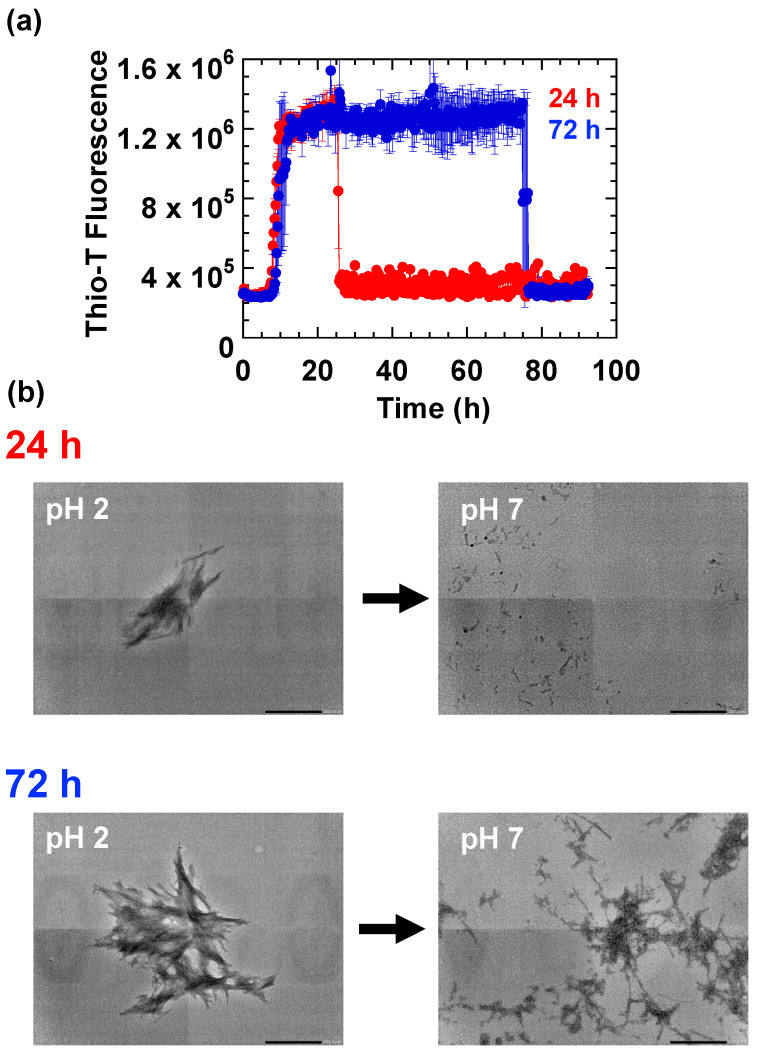
Extended incubation of HdeB fibrils at pH 2 leads to the appearance of a fibril form that can not be resolubilized at pH 7. (**a**) Time course of Thio-T fluorescence change. Two sets of experiments were performed in parallel. In the first set, samples of HdeB were incubated at pH 2.0, 250 mM NaCl to induce the formation of fibrils and subsequently shifted to pH 7.0 after 24 h. In the second set, HdeB was allowed to form fibrils at pH 2.0 as in the first set, but the samples were incubated (with shaking) for a further 48 h before adding Tris base to shift the pH to 7.0. For each set, representative aliquots were sampled before and after addition of Tris base to induce pH shift, and TEM images from these samples are shown in (**b**). Images were obtained at a magnification of ×20,000 (scale bar; 500 nm). Traces are averages ± standard errors of four separate samples obtained in two separate experimental sessions.

As shown in Figure 3, when samples of HdeB that were allowed to form fibrils at pH 2 were shifted to pH 7 after an interval of 24 h at acidic pH, the rapid decrease in Thio-T fluorescence was accompanied by the loss of observable fibril forms in the TEM images, leading us to believe that HdeB fibrils were indeed resolubilizing upon a shift in pH to 7 (Figure 3b, “24 h”). This rapid decrease in Thio-T fluorescence upon pH shift to 7.0 was also observed in samples that were incubated at pH 2.0 for a more extended interval (72 h; Figure 3a, blue). However, when we shifted the pH of these prolonged-incubation (72 h) HdeB samples to pH 7 and observed aliquots using TEM, we found that a considerable amount of fibrillar material remained visible in the TEM images (Figure 3b,”72 h”). From this experiment, we suspected that, upon prolonged incubation in low pH, HdeB fibrils that were initially “reversible”, i.e., resolubilized at pH 7 (hereafter denoted “Form I” fibrils) gradually transformed into a separate conformation that retained its fibrillar structure at pH 7.0, an irreversibly formed fibril state (“Form II” fibrils). Form II fibrils at pH 7.0 did not display a strong Thio-T fluorescence signal characteristic of other, typical fibril forms (Figure 3b, after 72 h), suggesting that the shift to neutral pH caused changes in morphology on the surface of Form II fibrils that translated to differences in Thio-T binding affinity.

In order to determine if this gradual transition of HdeB fibrils could be attributed to conformational changes within intact HdeB, rather than an artifactual effect caused by acid degradation and modification of the HdeB polypeptide by prolonged incubation at acidic pH, we analyzed the HdeB samples used in the fibrillation experiments using MALDI-TOF mass spectral analysis (Supplemental Figure S1). The experiments showed that neither soluble samples of HdeB incubated for 72 h at pH 2, nor fibril samples formed in pH 2, contained detectable amounts of degraded polypeptide. This result supported our idea that HdeB was forming fibrillar structures at pH 2 that gradually transformed into irreversible fibril forms upon prolonged incubation.

### 2.3. HdeB Fibrils Formed from Preformed Seeds Are Irreversible, Suggesting That Fibril Structural Traits Are Inherited

**Figure 4:**
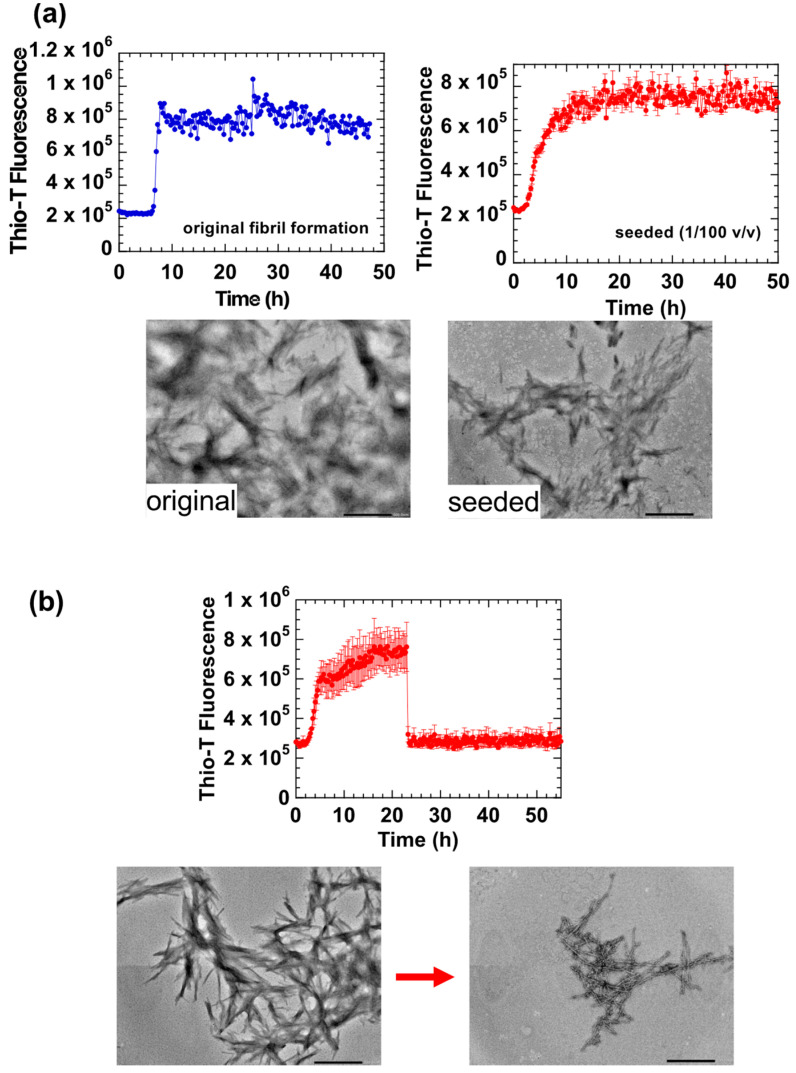
Seeded fibrillation experiments of HdeB performed at pH 2.0. (**a**) Seeds of “Form II” HdeB fibrils shorten the apparent lag phase of the fibril forming reaction, resulting in the production of “Form II’” fibrils. Initially, HdeB was incubated with shaking at pH 2.0 for 48 h (left, blue trace) to create Form II fibril samples. Next, an aliquot of this sample was added to freshly prepared acid denatured HdeB samples (1/100 *v*/*v* seeded assay) and fibrillation was allowed to proceed under the same conditions (right, red trace). Note the shorter lag time of the red trace compared to the original blue trace, which may be attributed to the presence of a fibril starter seed. Additionally, note that the left blue trace is the raw data of one sample that was subsequently used to provide seeding samples for the right red trace, and is therefore not an averaged data trace. TEM images of representative aliquots sampled at 48 h for each trace are shown below the time course panels. Images were obtained at ×20,000 magnification (scale bar; 500 nm). (**b**) pH shift experiments of Form II’ HdeB fibril samples. Form II’ HdeB samples were prepared and monitored for fibril formation according to the method used to obtain the red trace in (**a**). The pH of each sample was subsequently shifted to 7.0 at t = 24 h by addition of Tris base. Samples for TEM analysis were taken before and after the pH shift to obtain the images shown below the trace panel. Note that a significant amount of fibril matter remained under conditions where Form II HdeB fibrils would resolubilize (after a 24 h interval at pH 2, c.f. Figure 3a, red trace).

A slow structural transition that converts pH reversible HdeB fibrils (Form I) into an irreversible form (Form II) upon extended incubation at pH 2 may involve structural alterations that are inheritable, that is, transferable to fibrils formed from preformed fibril seeds. To determine if this was the case, we performed seeding experiments by adding preformed HdeB fibril samples as seeds to freshly prepared HdeB. We first prepared HdeB fibril samples at pH 2.0, and fibril development proceeded along a typical time course with a lag phase duration estimated as ~6 h (Figure 4a). Next, when we added a 1/100 *v*/*v* aliquot of this original fibril experiment to fresh HdeB preparations and repeated the experiment (Figure 4b), we observed a modest shortening of the lag phase (from ~6 h to ~3 h, compare the left blue trace representing the original fibril forming reaction with the right *red* trace representing the seeded reaction in Figure 4a) in the new samples that could be attributed to seed addition. When we probed the reversibility of these seeded fibrils by shifting the sample pH to 7.0 (Figure 4b), we noticed that, despite the short interval (24 h) that these seeded fibril samples were maintained at pH 2.0, and the immediate loss of Thio-T signal upon shift to pH 7.0, significant amounts of fibrillar aggregates were observed in the TEM image. This result supports the idea that HdeB fibrils that are formed by extension from preformed HdeB seeds (Form II’ fibrils) inherit, at least with regard to the reversibility of the fibrillation, structural characteristics of the preformed seeds, in this case Form II fibrils that were formed after 48 h at pH 2 (Figure 4a, blue trace).

### 2.4. Fibrils That Are Stable at pH 7 May Be Formed from a More Extensively Denatured, Disulfide Bond-Reduced Form of HdeB

From our experiments shown in Figure 1, Figure 2, Figure 3 and Figure 4, we deduced that fibrils resembling amyloid fibrils could be formed from monomeric acid-denatured HdeB under strongly acidic conditions below pH 3. The fibrils that were initially formed at pH 2 (Form I) were reversible and resolubilized at pH 7, but subsequently underwent slow structural rearrangements upon prolonged incubation while in the fibril state (Form II). In order to obtain insights regarding the specific structural characteristics that may be involved in these slow structural rearrangements, we became interested in finding experimental conditions that could circumvent this slow rearrangement and directly access the “post-conversion” state of acid-induced fibril that could not be resolubilized at pH 7.

Subunits of HdeA and HdeB both possess a single disulfide bond in its native state; in the case of HdeB, this disulfide bond is formed between Cys10 and Cys58 of the mature (signal-processed) sequence [4]. In the case of HdeA, reduction of the disulfide bond causes a large decrease [14] (but apparently not a complete loss [15]) of molecular chaperone activity, suggesting that the acid denatured structure is perturbed significantly by the loss of the disulfide bond. Although analogous data regarding the role of the disulfide bond on the structure and function of HdeB are not available, we wished to see if removing this bond would result in a more extensively unfolded, dynamic polypeptide whose fibril forming tendencies would be altered. Accordingly, we performed HdeB fibrillation experiments at pH 2.0 in the presence of 0.5 mM Tris(2-carboxyethyl)phosphine hydrochloride (TCEP-HCl), a reagent capable of reducing disulfide bonds under acidic pH conditions.

**Figure 5:**
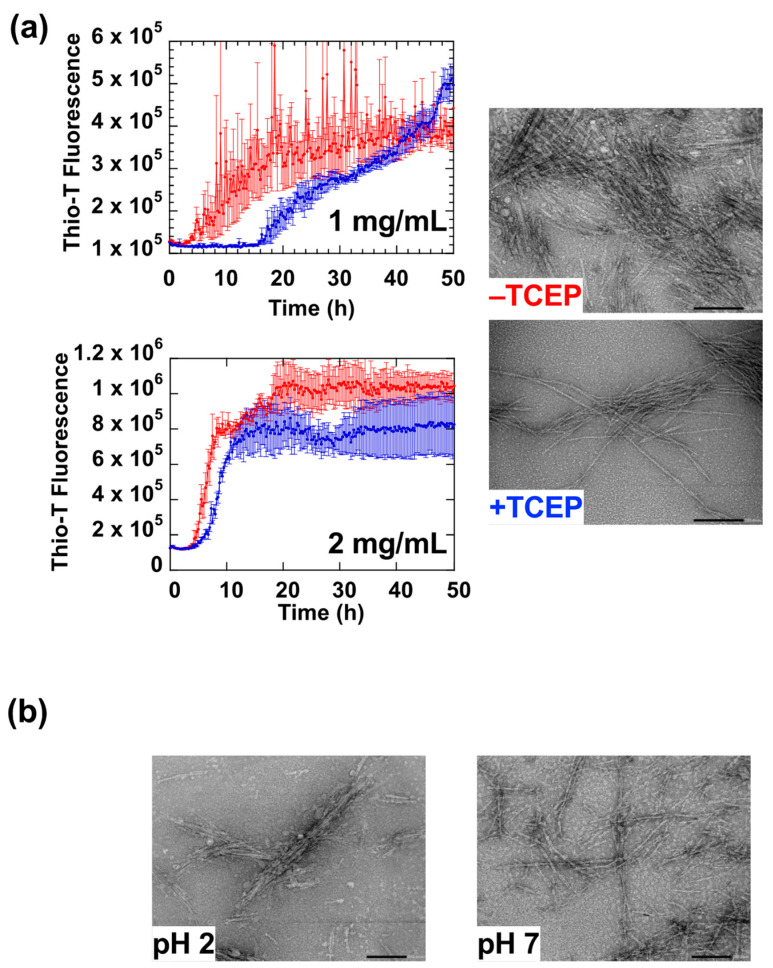
Fibril formation of reduced HdeB at pH 2. (**a**) Fibrillation at different concentrations of HdeB demonstrate the concentration dependence of the initial nucleus-forming lag phase. The upper panel shows assays performed at a protein concentration of 1 mg/mL, and the lower panel shows experiments performed at a protein concentration of 2 mg/mL. Red traces in each panel denote experiments with disulfide-intact HdeB, and blue traces denote experiments with 0.5 mM TCEP added to reduce the Cys10-Cys58 disulfide bond. Note the shortened lag phase in the blue traces at higher HdeB concentrations. To the right of the traces are representative TEM images of HdeB fibrils formed in the absence and presence of TCEP. Aliquots were taken from experiments shown in the upper panel (performed at 1 mg/mL). Images were taken at a magnification of ×50,000; scale bars denote 200 nm. (**b**) Fibrils of reduced HdeB formed at pH 2 are resistant to resolubilization at pH 7.

Figure 5a shows a comparison of the HdeB fibrillation reaction in the presence and absence of 0.5 mM TCEP. Adding TCEP to the reaction mainly resulted in an extension of the lag phase, suggesting that reduced HdeB required more time to form the initial fibril cores. This concentration dependence was confirmed in experiments shown in the lower panel in Figure 5a, where we increased the concentration of HdeB. When the HdeB concentration was doubled (to 2 mg/mL), the lag phase was shortened, a clear demonstration of the concentration-dependent characteristic of the initial fibril core forming reaction by reduced HdeB. In contrast, the rate of fibril extension, as estimated by the slope of Thio-T fluorescence increase after the lag phase, seemed to be only moderately affected by TCEP addition. Morphologically, TEM analysis of fibrils formed in the presence of TCEP tended to be longer with more regular repetitive features than fibrils formed in the absence of the reducing agent (Figure 5a, right).

Next, we performed experiments to probe the reversibility of the fibrils formed by reduced HdeB. As shown in Figure 5b, when fibril samples initially formed at pH 2 were shifted to pH 7.0, TEM analysis found that significant fibril matter remained at pH 7.0. This supports the idea that the fibrils formed by reduced HdeB (hereafter denoted “Form III” fibrils), which are more regular in morphology and formed more slowly, were resistant to resolubilization at pH 7.0; i.e., are a type of irreversible fibril. Due to the scission of the disulfide bond, HdeB was slow to aggregate into fibril seeds initially, but once formed, the resultant Form III fibrils resembled typical amyloid fibrils, long fibers with regular repeating structural features.

### 2.5. Secondary Structural Elements of HdeB Fibrils and Resolubilized Samples

In order to obtain additional information regarding the secondary structure of the numerous forms of HdeB fibrils that were detected, we performed attenuated total reflection Fourier transform infrared spectroscopy (ATR FT-IR) analyses on Form II and Form III fibrils, both before and after shifting the pH to 7.

**Figure 6:**
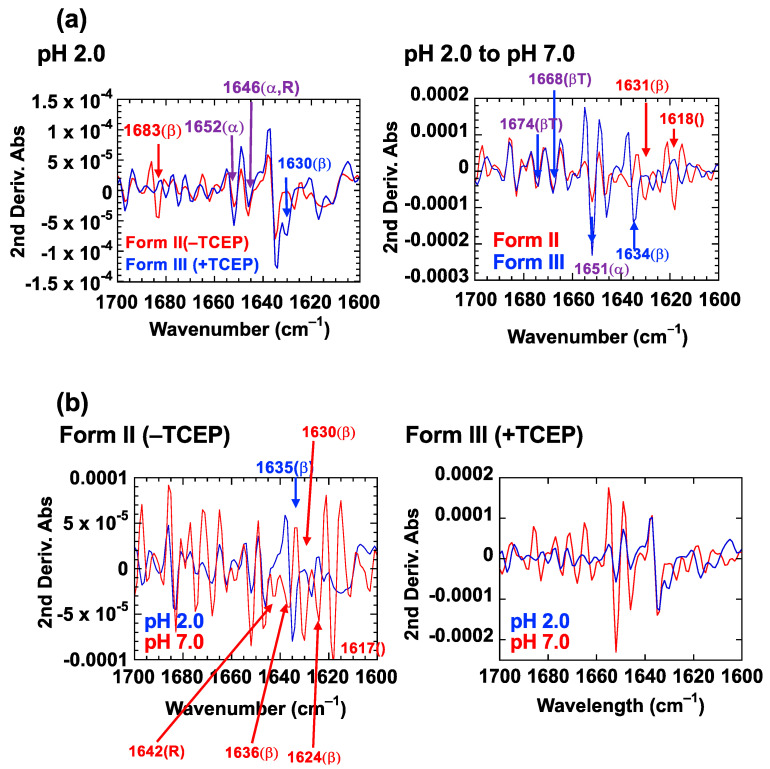
Secondary derivative analysis of ATR FT-IR spectra obtained from samples of Form II and Form III HdeB fibrils measured in D_2_O. HdeB fibrils were formed in the absence (Form II) and presence (Form III) of TCEP at pH 2, and aliquots were taken from each of these samples and mixed with Tris base to adjust the pH of the aliquots to 7. These four samples were then lyophilized for 48 h to remove the water contained. Each sample was applied to the FT-IR spectrometer and 4 µL of deuterium oxide was directly applied to rehydrate the sample. The IR spectra of the samples were then measured. In (**a**), Form II and Form III samples are compared both before (pH 2) and after (pH 2 to pH 7) shifting the pH to 7. In (**b**), changes in FT-IR spectra induced by pH shift are probed for Form II and Form III samples, respectively. The graphs correspond to second derivative spectra of the IR absorbance spectra in the 1700 cm^−1^ to 1600 cm^−1^ Amide I region. Distinctive minima (denoting the wavenumber positions of prominent IR absorbance peaks) are highlighted according to the sample attributed. In (**a**), red traces and notations of wavenumber correspond to Form II HdeB samples, blue traces and notations of wavenumber correspond to Form III fibril samples. Magenta wavenumber notes indicate minima observed similarly in both samples. In Panel (**b**), blue traces and notations of wavenumber correspond to samples maintained at pH 2 up until the lyophilization step, and red traces and wavenumber notes correspond to samples where the pH was adjusted to 7 using Tris base prior to lyophilization.

Figure 6 compares the second-derivative ATR FT-IR spectra of HdeB fibrils formed in the presence and absence of TCEP, in both the fibrillar (pH 2) and pH-neutralized (pH 2 to pH 7) forms. It should be noted that, due to the time required to prepare the samples for analysis, which involves prolonged incubation of samples at pH 2.0, the comparisons shown here are between the “Form II” and “Form III” fibrils, both of which are incapable of resolubilizing completely at pH 7. A total of four distinct spectra are compared according to two groupings; in (a), the effects of cutting the intrinsic disulfide bond are probed by comparing the spectra of Form II and Form III fibrils at pH 2 (left) and pH 7 (right). In (b), the effects of pH shift on the respective samples are probed through comparison of the fibrillar (pH 2) and pH neutral (pH 7) samples.

From Figure 6a left, we deduce first of all that both Form II and Form III fibrils retain significant α-helical content, as evidenced by the characteristic secondary derivative minima at 1652 cm^−1^ and 1646 cm^−1^ (noted in magenta) [16]; we note however that it is possible that the signal at 1646 cm^−1^ represents random structure instead of α-helix structure. Spectra of both Form II and Form III fibrils also showed minima that could be attributed to β-structure. However, the values of the minima seen for the two samples were at different frequencies; spectra of Form II fibrils showed a minimum at 1683 cm^−1^, whereas spectra of Form III fibrils showed a minimum at 1630 cm^−1^. These differences suggested that subtle structural differences are present in the secondary structure of the two fibril forms, mainly in the beta component. This observation may be indirectly supported by the differences that we detected in fibril morphology observed using TEM (Figure 5a).

A comparison of spectra from the respective fibril forms after the shift to pH 7.0 showed additional complexities (Figure 6a, right). As seen in this panel, the two HdeB fibril samples at pH 7.0 both displayed common minima at 1674 cm^−1^, 1668 cm^−1^, and 1651 cm^−1^, which suggested the presence of residual β-turn and α-helical structures. At the same time, the two spectra also showed prominent differences, most notably in the lower wavenumber region. The specific values of the minima detected were in each case suggestive of the presence of beta structure, but the specific wavenumber that was detected was different in the two spectra. Taken together, the comparison of FT-IR second derivative spectra suggested that, depending on the initial condition of the acid-denatured HdeB sample, both the fibrils that are formed at low pH as well as the residual structures that remain after adjusting the pH of the samples to 7.0 have distinct structural characteristics that distinguished Form II fibrils from Form III fibrils. As traits common to both Form II and Form III fibrils, each spectra showed evidence of significant alpha helical structure as well as beta structure, and the differences in characteristics were mainly reflected in the specific wavenumber values corresponding to beta structure.

Next, we focused on the differences in the two fibril forms (±TCEP) with regard to the reversibility of the fibril forming reaction, by comparing the spectra of HdeB samples formed before and after pH shift to 7. The most prominent difference between Form II (Figure 6b left) and Form III (Figure 6b right) fibrils was that, changes in spectra that were triggered by pH adjustment to 7.0 were much less obvious in Form III fibrils, compared to Form II fibrils. We deduce that Form III HdeB fibrils, in other words, formed from HdeB without the disulfide bond, are much more resilient to pH change compared to Form II fibrils, formed from HdeB with the disulfide intact.

## 3. Discussion

In this study we characterized the fibril forming characteristics of HdeB, a periplasmic molecular chaperone from *E. coli* which utilizes an acid-denatured conformation to express molecular chaperone activity. Along with its companion chaperone, HdeA, HdeB represents an interesting example of a denatured protein conformation fulfilling a function that is integral to cellular viability under stressful conditions. The prevalence of intrinsically denatured proteins in extensive and complex biological mechanisms in eukaryotes [17], as well as the importance of intrinsic disorder in certain functional systems of prokaryotes [18], have demonstrated the importance of observing and understanding the expanded structural landscape of protein structure and function encompassing both folded and unfolded states. HdeA and HdeB are an interesting example of the way in which protein dynamism may be utilized to achieve additional regulatory functions (in this case, pH stress sensing) that aid cells in maintaining integrity. Additionally, protein fibrillation and deposition, often correlated with the onset of numerous diseases, are phenomena that are also highly relevant to this protein structural dynamism, as is the presence and actions of molecular chaperones that shepherd the dynamic proteome. HdeB (and HdeA), by virtue of showing all of these myriad facets of protein structure and dynamism in a single entity, provides a window through which this subject may be studied in detail.

### 3.1. Conditionally Reversible Fibril Formation of HdeB and Formation of Multiple Fibril Forms

Based upon an initial assumption that HdeB would be susceptible to fibrillation in its active, acid-denatured form, in a manner similar to that seen for HdeA, we performed experiments that confirmed this assumption: HdeB was indeed readily converted into fibrillar aggregates from the acid-denatured state (Figure 1). The specific pH where fibrillation was observed (pH < 3, Figure 1a) corresponded to conditions where the HdeB dimer denatures and dissociates into monomers, and is inactive as a molecular chaperone [5,12]. The pH range at which fibrillation was observed was similar to the conditions where HdeA tended to form fibrils, as determined in a previous study [13]. Functionally, comparing the characteristics of HdeA and HdeB fibrillation is interesting, since evidence suggests that for HdeA, there might be a certain amount of overlap between conformations that are important for molecular chaperone activity and conformations that were prone to form fibrils, resulting in a competitive mechanism of sorts. For HdeB, molecular chaperone activity is linked to the retention of the native dimeric state, and so fibril formation would not be fundamentally linked to its molecular chaperone activity in a way that is seen for HdeA. This reversible fibrillation was observed under a range of HdeB concentrations, demonstrating that fibrillation proceeded according to a process involving an initial nucleation of fibril cores that was protein concentration dependent, followed by rapid extension of fibrils (Figure 2). The specific mechanism of fibrillation remains to be determined.

Interestingly however, we found that in the case of HdeB, numerous experimental conditions could be selected that resulted in this reversibility of the fibril forming reaction to be challenged, resulting in the detection of multiple alternate fibril forms (Form II, II’ and III) each of which retained their fibrillar structure at pH 7. Extended incubation at pH 2, seeding with preformed irreversible seeds, or formation of fibrils from reduced HdeB each led to the production of an alternate fibril forms that retained its fibril structure at neutral pH. Compared to HdeA, it seemed that in the case of HdeB, various residual structures that remain or were formed under each experimental condition can lead to alternate forms of HdeB fibrils. Some of these forms, such as Form II and Form III, were distinguishable at the secondary structural level. Although differences in fibril morphology under various experimental conditions were also observed for HdeA [13], the structural landscape of HdeB with regard to the acid denatured states that lead to formation of fibrils seem to be much more diverse and complex compared to HdeA. A more detailed analysis of the fibrillation kinetics will lead to additional information regarding the structural and kinetic principles that govern these various fibril forms of HdeB, and also the relationship between these multiple fibril forms.

**Figure 7:**
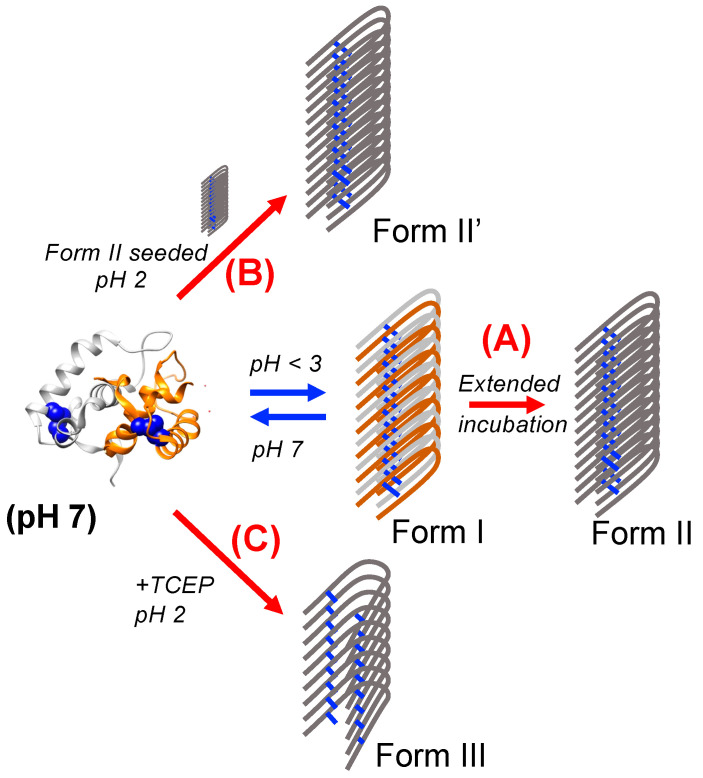
Schematic illustration of the results described in the present study. HdeB (dimeric, disulfide intact) may be induced to form regular fibrils (Form I) at pH conditions lower than 3 (Blue arrow). This original Form I fibril is capable of resolubilizing upon pH shift to 7. Certain alternate protocols (Red arrows) result, respectively, in the formation of irreversibly formed HdeB fibrils that remain stable at pH 7. (**A**) Extended incubation at low pH results in formation of Form II fibrils (disulfide intact, irreversible). (**B**) Form II fibrils may be used as seed to induce the direct formation of irreversible Form II’ fibrils. (**C**) Incubation with reducing agent TCEP during fibril formation at pH 2 induces the formation of irreversible Form III (disulfide deduced, irreversible) fibrils. Forms II and III are distinguishable through FT-IR analysis (Figure 6); at present, we lack evidence to discriminate between the Form II and Form II’ fibril forms. The dimeric backbone image of HdeB at pH 7 was produced from coordinates derived from PDBID 2xuv [5] using UCSF Chimera [19].

Figure 7 is a schematic summary of the results in the present study. We determined that the periplasmic chaperone HdeB could form fibrillar structures resembling amyloid fibrils from an acid-denatured state. The initial fibrillar form is capable of resolubilizing from fibrils at pH 7 (Figure 7, blue arrows), but numerous alternative fibril forms were also created under a variety of conditions, all of which were incapable of recovering the soluble state at pH 7 ((A)~(C), red arrows). As outlined in Figure 7, at present, our data on HdeB reflect a very complex fibrillation reaction that is sensitive to the starting conditions of the polypeptide, the conditions under which fibrillation occurs, and the reaction time. We have not addressed the implications that such complex fibrillation reactions pose to the structure–functional relationship of HdeB in the actual *E. coli* periplasm, but we note that the actual environment of the periplasm includes numerous additional factors such as periplasmic proteins and lipids that could conceivably alter the behavior of HdeB in subtle ways, especially when we consider the biological activity of HdeB as an acid-sensitive molecular chaperone. Further probes will be necessary to deduce the specific structural details that underlie this complexity.

### 3.2. Possible Biological Relevance of the Fibril Forming Reaction of HdeB

A prominent characteristic of the fibril forming reaction of HdeB shown in our experiments was that fibrillation was promoted under conditions where the molecular chaperone activity of HdeB was expected to be inactive (pH < 3) [5,12]. This characteristic was in contrast to that of HdeA observed in a previous study, where fibrillation was in direct competition with the molecular chaperone activities [13]. Since the behavior of HdeB characterized in this study should therefore be regarded as an event unrelated to its biological function, a question arises: what are the possible biological implications of HdeB fibrillation at low pH?

**Figure 8:**
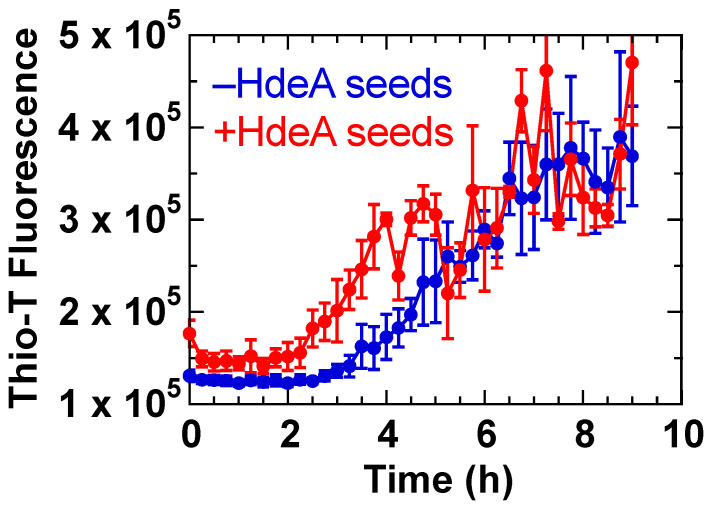
Preformed seeds of HdeA promote the fibrillation of HdeB at pH 2. Mixing a 1/100 (by mass) aliquot of preformed HdeA fibril sample to soluble HdeB at pH 2 resulted in a shortened lag phase during the fibril forming reaction of the latter protein, indicating a heterogeneous stimulating effect by HdeA seeds on HdeB fibrillation. Since HdeA and HdeB are both susceptible to forming fibrils under these conditions, these examples of “cross-seeding” events suggest implications that are relevant to the actual conditions inside the *E. coli* periplasm.

Although we were unable to obtain from our experiments conclusive evidence regarding the relationship between HdeB fibrillation and its biological activities in the *E. coli* periplasm, one possible consequence of HdeB acid-induced fibrillation might involve interactions between HdeA and its fibrils. Since the conditions under which HdeA and HdeB form fibrils are nearly identical and the two proteins typically occupy the same space in the *E. coli* periplasm, it was conceivable that these two molecular chaperones would affect each other when the periplasm undergoes a shift to strongly acidic conditions (pH < 3). To determine if this was a probable occurrence, we performed HdeB fibrillation reactions in the presence of a small amount of preformed HdeA fibril seeds. As seen in Figure 8, we found that seeds of HdeA were able to promote the fibril formation of HdeB, suggesting that heteromolecular interactions between these two proteins could conceivably occur in the periplasm under acidic conditions to alter their structural behavior. The periplasm is a highly heterogeneous space containing various proteins, peptidoglycans, and membrane associated hydrophobic components, and interactions between HdeB and these components would be commonplace. Through a highly sensitive and extensive chemical crosslinking and proteomic assay, Zhang and colleagues have identified the various client proteins in the periplasm that are recognized and bound by HdeA and HdeB at pH 2.3 [7]. Their results revealed that HdeA and HdeB recognize a variety of membrane bound and soluble protein clients, including some molecular chaperones that are responsible for the well-being of other periplasmic proteins. The extensive data provided by Zhang et al. suggest that the conditions in the periplasm involve numerous intermolecular interactions between various components, each possibly altering the functional characteristics of each other in complex ways. Interestingly, with regard specifically to HdeA and HdeB, Zhang et al. also documented a synergetic effect between the molecular chaperone activities of HdeA and HdeB against the client protein SurA (a periplasmic molecular chaperone) [7]. This result was an interesting example of the possible cooperative interplay between HdeA and HdeB under severely acidic conditions that would produce unexpected additional activities, since HdeB would not be expected to act as a molecular chaperone under the conditions studied by Zhang et al. (pH 2; [7]). Our results in Figure 8, as well as the results of Zhang et al. reflect the inherent complexity of the periplasmic environment, and we intend to carefully probe these complex interactions in future efforts to shed light upon the dynamic aspects of the periplasmic proteome.

## 4. Materials and Methods

### 4.1. Vectors and Protein Purification

The expression vector pET-HdeB was constructed in a manner similar to that used in the construction of pET-HdeA, by combining the vector fragment of the overexpression vector pET-EL with a synthesized gene (GeneArt™, Thermo Fisher Scientific K. K., Tokyo, Japan) of the HdeB precursor containing the periplasmic transport signal peptide sequence. Briefly, the vector and insert DNA fragments were amplified from template using PCR with Prime Star HS DNA polymerase (Takara Bio K. K., Shiga, Japan), and the primers used in the amplification incorporated a 15 nt overlap at the ends. The amplified DNA was used to construct the final circular vector by using the InFusion HD Cloning Kit with Cloning Enhancer (Takara Bio K. K., Shiga, Japan). The vector was sequenced in order to confirm that the product contained the desired sequence.

Competent BL21(DE3) cells were transformed chemically using pET-HdeB to obtain *E. coli* cells overexpressing HdeB. Cells were cultivated in LB broth, pH 7.4 at 37 °C. Cultivated cells (12 g in a typical purification session) were washed twice with 100 mL 0.2 M NaCl, and the wash solution containing mature HdeB was recovered, concentrated using centrifugal ultrafiltration, and dialyzed against 30 mM Tris-HCl (pH 8.0 at 4 °C) to obtain crude HdeB samples. This sample was next purified on a Resource-Q anion exchange column using an AKTA-FPLC system (Cytiva, Tokyo, Japan). Proteins were eluted using a linear gradient of 0 to 0.5 M NaCl; HdeB fractions were typically eluted at 0.1 M NaCl. The anion exchange step was repeated under identical conditions to obtain purified HdeB. Purified protein samples were quantitated using the Bradford method and the Bio-Rad protein assay kit and stored at 4 °C until use (Bio-Rad Laboratories, Hercules, CA, USA). Typically samples were used within 1 mo of purification.

### 4.2. Fibril Forming Assays of HdeB Monitored by Thio-T Fluorescence

Assays of fibril formation using the specific binding and characteristic fluorescence of Thio-T were performed in 20 mM Gly-HCl buffer, according to previously reported protocols used to monitor HdeA fibrillation [13]. The concentration of Thio-T during assays was set to 20 µM, and HdeB concentrations, were set to 1 mg/mL unless stated otherwise (Figure 2 and Figure 4). 150 µL samples were prepared in 96-well fluorescence plates (Greiner Bio-One Fluotrac µClear, Griener Bio-One, Tokyo, Japan) and monitored intermittently using a Perkin-Elmer ARVO-X4 plate reader (Waltham, MA, USA), using the agitation ability of the instrument to induce shaking. In pH shift experiments where acidic samples were mixed with 2 M Tris-base solution to modify the pH to 7, mock preparations were prepared and used to estimate the proportional volume of Tris base needed to achieve the desired results, and the pH of the samples were also measured after the assay to confirm that the shift to pH 7 had been achieved.

In the experiments shown in Figure 8 where we added preformed seeds of *E. coli* HdeA to soluble HdeB samples, HdeA fibrils were first prepared in 20 mM Gly-HCl buffer, pH 2, containing 250 mM NaCl by shaking at 37 °C for 24 h. the concentration of HdeA was 1.0 mg/mL. A 1.5 µL aliquot of this preformed fibril sample was then added to 150 µL of HdeB (1.0 mg/mL) samples freshly prepared in the same buffer. Fibril formation was then monitored as described above using Thio-T. We also monitored the reaction of the initial HdeA fibrillation to confirm that HdeA indeed formed characteristic fibrils under the conditions used [13].

### 4.3. TEM

Five microliter aliquots of various samples measured for fibril formation as described in *4.2* were taken, diluted 20- to 30-fold with fresh buffer that matched the conditions for each sample and applied to collodion-covered carbon mesh electron microscopy sample grids (Nisshin EM Co., Tokyo, Japan). After 90 s, the aliquots were removed from the grids, washed with 5 µL Milli-Q and negatively stained using EM-Stainer solution. TEM micrographs were taken on a JEOL 1400Plus electron microscope (Tokyo, Japan) operating at 80 kV.

### 4.4. ATR FT-IR Analysis

ATR FT-IR spectra were measured as previously described on a Perkin-Elmer Spectrum 65 FT-IR spectrophotometer (Waltham, MA, USA) equipped with the universal ATR sampling accessory (Diamond/ZnSE crystal). Samples of HdeB prepared under various conditions in their respective buffers were lyophilized for approximately 48 h to remove water. Measured samples were first placed directly onto the instrument, followed by application of 2 µL 99.9% deuterium oxide (D_2_O, Cambridge Isotope Laboratories, Tewksbury, MA, USA). Samples were measured after a 5 min incubation. Second derivative spectra were obtained by using the software package (Spectrum ver.10.03.09) provided with the instrument (Waltham, MA, USA).

## Data Availability

All data are available upon request to the corresponding author (T.M.).

## Supplementary Materials

The following supporting information can be downloaded at: https://www.mdpi.com/article/10.3390/ijms232113243/s1. Reference [20] is cited in the supplementary materials.
