# Supplementary material for: Formation of Fibrils by the Periplasmic Molecular Chaperone HdeB from Escherichia coli
Source: Int J Mol Sci. 2022 Oct 31;23(21):13243. doi: 10.3390/ijms232113243 (PMC9657021; doi:10.3390/ijms232113243)

Figure S1; MALDI-TOF Mass spectrum analysis of HdeB samples.

Mass spectra analyses were performed on a Bruker ultrafleXtreme instrument. Samples of HdeB (1 mg/ml) were diluted tenfold with 0.1% trifuoroacetic acid and mixed with an equal volume of matrix solution (a saturated solution of alpha-4-cyano-4-hydroxycinnamic acid dissolved in acetonitrile:0.1% trifluoacetic acid=30:70 solvent). After mixing, samples were loaded onto ground steel target plates, and allowed to dry before loading into the instrument for analysis. Analysis was performed in Linear Positive mode, 1~20kDa, with an acceleration voltage of 20 kV. Peaks were calibrated using the Protein Calibration Standard I provided by the manufacturer.

Panel (a): Native HdeB stock solution stored at 4°C in pH 7 Tris buffer. Panel (b): HdeB incubated at 37°C for 72 h in 20 mM Gly-HCl buffer, pH 2, containing 250 mM NaCl. Panel (c): samples of HdeB that were allowed to form fibrils at pH 2 by shaking and incubation for 72 h in 20 mM Gly-HCl buffer, pH 2, containing 250 mM NaCl. There were no additional peaks that could be assigned to degraded protein peptides in any of the samples analyzed. The estimated molecular weight of the mature HdeB polypeptide is 9065 (Reid, G. E., et al. (2002).J. Am. Chem. Soc. **124**(25): 7353-7362).

(a)

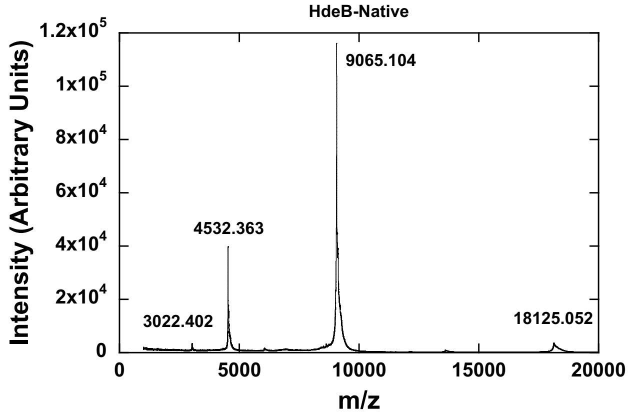

(b)

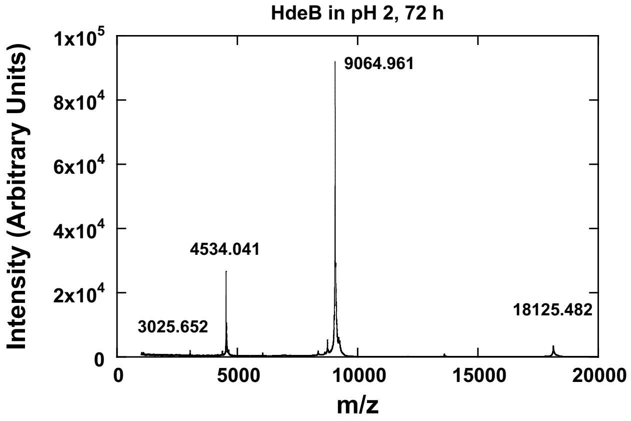

(c)

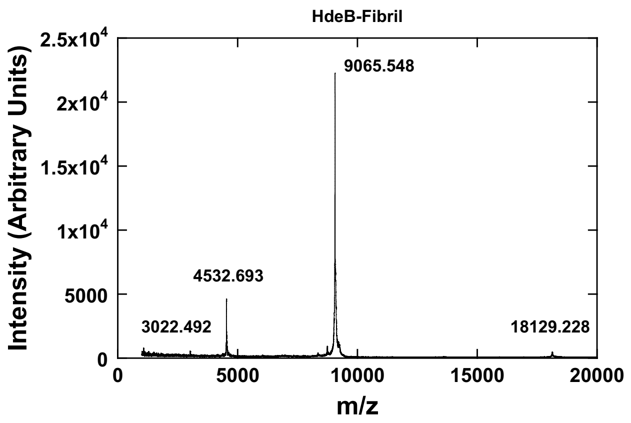

Supplement: Supplementary file 1 [file ijms-23-13243-s001.zip › ijms-1870077-supplementary.pdf]
